# The Influence of Temperature Changes on the Rice Starch Structure and Digestive Characteristics: One and Two-Step Annealing

**DOI:** 10.3390/foods11223641

**Published:** 2022-11-14

**Authors:** Guiyuan Xiang, Jiangtao Li, Wenfang Han, Yaqin Yang, Qinlu Lin, Ying Yang, Qiongxiang Liu, Xiaofeng Guo, Qianru Pan, Zhengyu Huang, Lingxue Cao

**Affiliations:** 1 National Engineering Research Center of Rice and Byproduct Deep Processing, College of Food Science and Engineering, Central South University of Forestry and Technology, Changsha 410004, China; 2 National Research and Development Center for Egg Processing, College of Food Science and Technology, Huazhong Agricultural University, Wuhan 430070, China; 3 Key Laboratory of National Forestry and Grassland Administration for Control of Diseases and Pests of South Plantation, Central South University of Forestry and Technology, Changsha 410004, China

**Keywords:** starch structure, annealing temperature, digestibility

## Abstract

This study investigated the effects of annealing on the structural and physicochemical properties of rice starch below the onset temperature (To) by 5 °C and 15 °C. The results revealed that annealing improved the gelatinization temperature of rice starch, decreased the swelling power, solubility, and paste viscosity of rice starch, and had no significant effects on the morphological structure and crystal configuration of rice starch. In one-step annealing, the annealing temperature of 60 °C is more conducive to the rearrangement of starch molecules, so its crystallinity, short-range ordered structure, and gelatinization temperature are higher than at 50 °C; however, its RDS, SDS, and RS contents will be increased. During the two-step annealing treatment, the temperature change is not conducive to the molecular chain rearrangement and to the formation of perfect crystalline structure, which increases the sensitivity of enzymes to starch, so the RDS content of starch increases significantly, while the RS content decreases.

## 1. Introduction

Starch is a semi-crystalline polymer composed of glucose, which is deposited in the form of granules in the roots, stems, and leaves of green plants and widely used in foods, medicines, and cosmetics [1,2]. However, natural starch is poor in functionality and prone to aging, which reduces the viscoelasticity of the product and causes poor gel stability and transparency [3,4]. Starch is generally modified by using physical, chemical, or enzymatic methods and their combinations to improve its specific functional properties. In food processing industrial applications, there is increasing attention on physical modification of starch that employs moisture, heat, radiation, or shear to modify starch without the potential risk caused by chemical modification [5].

Annealing of starch is performed in excessive (>65% *w*/*w*) or appropriate (40–55% *w*/*w*) moisture and at temperatures above glass transition and slightly below gelatinization [6,7,8]. Annealing can greatly modify the functional properties of starch without destroying its granular structure, form a more stable configuration, and reduce amylose leaching by promoting the reorganization of starch molecules [9]. It is also possible to change the crystallization characteristics of starch by promoting the rearrangement of molecular chains within starch to cause the growth of starch crystals [10]. Compared with natural starch, the thermostability, gelatinization temperature, acid resistance, and shear resistance of starch after annealing are enhanced [11,12,13]. In the process of the annealing modification of starch, the ratio of starch to water, the temperature of annealing, and the heat treatment time are the key parameters that need to be controlled [14]. Liu et al. [15] found that annealing at 30 °C has a more obvious effect on the granular morphology of corn starch than annealing at 50 °C. Additionally, it has also been reported that through continuous and repeated annealing, the modified starch obtained by the repeated annealing has better functional characteristics and can be used as an effective treatment method for the production of modified functional starch with different industrial applications [16,17]. However, starch is modified at a single annealing temperature or by repeated annealing at a single temperature in the most cases, and few reports have been found on continuous annealing at different temperatures.

Based on current researches [16,17,18], this paper proposes a hypothesis that the structural and physical properties of starch annealed at multi-step temperatures will be significantly different from that annealed at a single temperature. Based on this hypothesis, this experiment uses rice starch as raw material, modifies rice starch through annealing treatment at a single temperature and at two-step temperatures, and explores the effects of annealing temperature and method on the structure, functional properties, and in vitro digestibility of rice starch, in order to provide a new research idea for annealing modified rice starch.

## 2. Materials and Methods

### 2.1. Materials

The rice in this study was obtained from Hall 4, Yupei, Nanling, Wuhu, Anhui, China. The rice starch was milled to 100−mesh. A glucose oxidase/peroxidase (GOPOD) was purchased from Megazyme International Ireland (Bray Business Park, Bray, Co., Wicklow, Ireland). Amyloglucosidase and α-amylase were purchased from Sigma-Aldrich Co. LLC, Santa Clara, CA, USA. All other reagents in the experiments were of analytical grade.

### 2.2. Preparation of Samples

#### 2.2.1. Rice Starch Preparations

The starch isolation from rice flour was based on Bian et al. [19], with minor modifications. Rice that had been washed 2 to 3 times with pure water was dispersed in NaOH solution (0.2%) and stirred continuously for 30 min after soaking at 25 °C for 4 h, and the NaOH solution was replaced every 4 h for a total of 24 h. The rice slurry was wet ground with a colloid mill and centrifuged at 4000r/min for 15 minutes. The yellow supernatant was discarded and the starch residue was washed repeatedly with pure water. After that, the purified rice starch was dried in an oven (40 °C), milled to 100−mesh, and named NRS.

#### 2.2.2. Annealing Treatment

The method was based on Xu et al. [17], with minor modifications. The onset temperature (T_O_) of the starch was determined by DSC at 65° in pre-experiments. The natural rice starch (25 g, dry basis) was dispersed in 0.02% sodium azide and 75 mL of distilled water and divide into 4 equal parts. The first sample was heated at 50 °C (T_O_ −15 °C) for 48 h, which was named ANN−50. The second sample was heated at 60 °C (T_O_ −5 °C) for 48 h, which was named ANN−60; the third sample was first heated at 50 °C for 12 h and then transferred to 60 °C for 12 h to obtain the two-step annealed starch, which was named ANN−50−60. The fourth sample was first heated at 60 °C for 12 h and then transferred to 50 °C for 12 h to obtain the two-step annealed starch, which was named ANN−60−50.

After annealing, the sample was cooled to 25 °C, followed by centrifugation at 4000 r/min for 15 min. The precipitation was collected, dried in an oven (40 °C), and milled to 100−mesh.

### 2.3. Polarized Light Microscopy (PLM) and Optical Microscope (OM)

A drop of starch solution with a mass fraction of 1% was placed onto the slide and covered with the coverslip. The morphologies of the samples were observed using an optical microscope (Nikon, Tokyo, Japan) under crossed-polarized light (magnification 200×). 

### 2.4. Scanning Electron Microscopy (SEM) 

The morphologies of the starch samples were observed using a SEM (JEOL, Tokyo, Japan), prepared for observation as described by Huang et al. [16]. 

### 2.5. X-ray Diffraction (XRD)

XRD analyses of the starch samples were determined by an XRD (Rigaku Corporation, Tokyo, Japan) equipped with CuKα radiation source (λ = 1.5406 Å, voltage 40 kV, and current 40 mA). The samples were scanned from 5° to 40° (2θ), at a 5° min^−1^ rate. The relative crystallinity (RC) was calculated using the Peakfit software with the following specific formula:

(1) $$\mathrm{RC}\%=\frac{\mathrm{Ac}}{\left(\mathrm{Ac}+\mathrm{Aa}\right)}\times 100\%$$

where Ac is the area of the crystalline region, and Aa is the area of the amorphous region [1].

### 2.6. Fourier Transform Infrared Spectroscopy (FT-IR)

The FT-IR spectra of the starch samples were based on Ji et al. [20], with minor modifications. The FT-IR spectra of starch samples were determined by using a FT-IR (IRTracer-100, Shimadzu, Japan). The rice starch samples were mixed with KBr (starch/KBr: 1/100, *w*/*w*), compressed, and scanned at a speed of 4 cm^−1^ in the range of 4000–400 cm^−1^; the number of scans was set to 64. After that, the original spectrum was deconvoluted using the Omnic 9.2 software.

### 2.7. Pasting Properties (RVA)

Starch samples (3.0 g, dry basis) were mixed with 25 mL of distilled water, and pasting properties were obtained by using a RVA-Super4(perten, Stockholm, Sweden); the program was set following the procedure of Hu et al. [21]. Peak viscosity (PV), setback (SB), final viscosity (FV), and breakdown (BD) were calculated from the RVA profiles.

### 2.8. Thermal Properties (DSC)

Thermal properties of the starch samples were obtained by using a differential scanning calorimeter (TA Instruments, New Castle, DE, USA). Starch samples were mixed with distilled water (1/3, *w*/*w*) and equilibrated overnight at 25 °C. Then, the samples were scanned at a heating rate of 10 °C min^−1^ from 20 to 110 °C, and the onset (T_O_), peak (T_P_), and conclusion temperatures (T_C_) and the gelatinization enthalpy change (ΔH) were recorded.

### 2.9. Swelling Power and Solubility

The swelling power (SP) and solubility (S) of rice starch samples were obtained according to the method of Xu et al. [17], with minor modifications. We dispersed the starch sample (0.5 g, dry weight) in a centrifuge tube containing 30 mL of distilled water and placed it in a water bath at 55, 65, 75, 85, and 95 °C for 30 min, respectively. The centrifuge tubes that were cooled to 25 °C were then centrifuged at 4000 r/min for 30 min, and the supernatant was dried in an oven at 105°C and weighed; the precipitate was immediately weighed. S and SP were calculated using the following formula:

(2) $$S\left(\%\right)=\frac{A}{W}\times 100\%$$

(3) $$\mathrm{SP}\left(\%\right)=\frac{P}{W\times \left(100-S\right)}\times 100\%$$

where *W*, *A*, and *P* represent the dry sample weight, supernatant weight, and precipitate weight, respectively.

### 2.10. In Vitro Digestibility

The digestibility of the starch samples was obtained according to the method of Englyst et al. [22], with minor modification. The sample (100 mg) was vortex-dispersed in 15 mL of sodium acetate buffer (0.2 mol/L, pH 5.2) and equilibrated at 37 °C for 5 min; then, 5 mL of a ready-to-use enzyme solution containing porcine pancreatic α-amylase (450 U/mg) and amyloglucosidase (51 U/mL) was added. The mixture was then immersed in a water bath at 37 °C and hydrolyzed at 160 r/min. The mixture (500 μL) was treated in absolute ethanol (4 mL) at intervals of 0, 5, 10, 20, 40, 60, 90, 120, and 180 min to inactivate the enzyme, followed by centrifugation at 4000 r/min for 10 min. After centrifugation, the supernatant was taken to measure the glucose content (Gt) using the GOPOD kit, and the hydrolysis rate, rapidly digestible starch (RDS), slowly digestible starch (SDS), and resistant starch (RS) of the sample were calculated according to the following formulas:

(4) Hydrolysis rate (%)= Gt ×0.9 /TS ×100% 

(5) RDS (%)=(G20−FG)×0.9 /TS ×100%

(6) SDS (%)=(G120−G20)×0.9 /TS ×100

(7) RS (%)=[TS−(RDS + SDS)]×0.9 /TS ×100%

where FG is the free glucose content of starch, and TS is the total starch weight.

### 2.11. Statistical Analysis

Unless otherwise noted, all results in this article were expressed as the mean ± standard deviation of three experiments at 95% (*p* < 0.05) using SPSS 20.0 (SPSS Inc., Chicago, IL, USA), by Duncan’s multiple test method. Plots were all performed using Origin 8.5 (Northampton, MA, USA).

## 3. Results

### 3.1. Morphology of Granules

**Figure 1:**
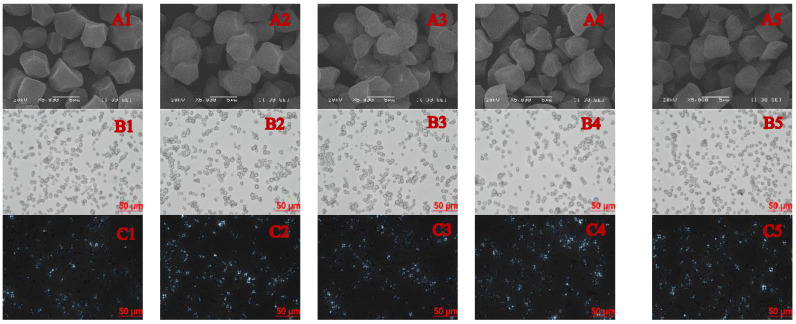
SEM images at 5000×magnification (**A**), OM images at 200×magnification (**B**), and PLM images at 200×magnification (**C**) of the one and two-step annealed rice starch samples. 1, NRS; 2, ANN-50; 3, ANN-60; 4, ANN-50-60; 5, ANN-60-50.

The granular microstructures of the rice starch samples are presented in Figure 1. Natural rice starch (Figure 1A) has granules with relatively small sizes, irregular polygonal shapes, and smooth surfaces without cracks and pores. The annealed starch had granules with smoother polygonal edges and more surface folds, which may be due to the swelling of the starch granules under certain hydrothermal conditions. In the one-step annealing, the folds present on the surface of the ANN−60 were more obvious, compared to the ANN−50, indicating that the annealing temperature close to T_O_ had a greater impact on the morphological structures of starch granules. The result was consistent with the findings of Wang et al. [9], who found that the annealing of wheat starch at 30 °C and 40 °C, respectively, did not influence the morphologies of wheat starch granules, but starch granule aggregation and destruction were noticed during the 50°C annealing process. 

Compared with one-step annealing, the surface folds of the two-step annealed starch granules (Figure 1(A4,A5)) were significant, of which the surface folds of ANN−60−50 were slightly more than those of ANN−50−60, possibly resulting from the temperature changes in the two-step annealing that aggravated the dissolution of amylose in the hydrothermal effect. Shi et al. [2] found that annealing at different temperatures and times had no effect on the morphology of starch from *Castanopsis sclerophylla*. These results showed that changes in the morphological characteristics caused by annealing modification might depend on starch source and treatment conditions. 

In the polarized light microscopy, the rice starch granules showed Maltese cross structure before and after annealing. The Maltese cross structure is related to a difference in density and refractive index between two different crystal structures and amorphous structures inside starch granules; the Maltese cross structure is formed when the polarized light passes through the starch granules [20]. The Maltese cross phenomenon of annealed rice starch is more obvious under polarized light microscopy, which may be due to the fact that annealing not only does not destruct the crystalline structure, but it also enhances the crystal structure integrity and stability of the starch granules.

### 3.2. XRD

**Figure 2:**
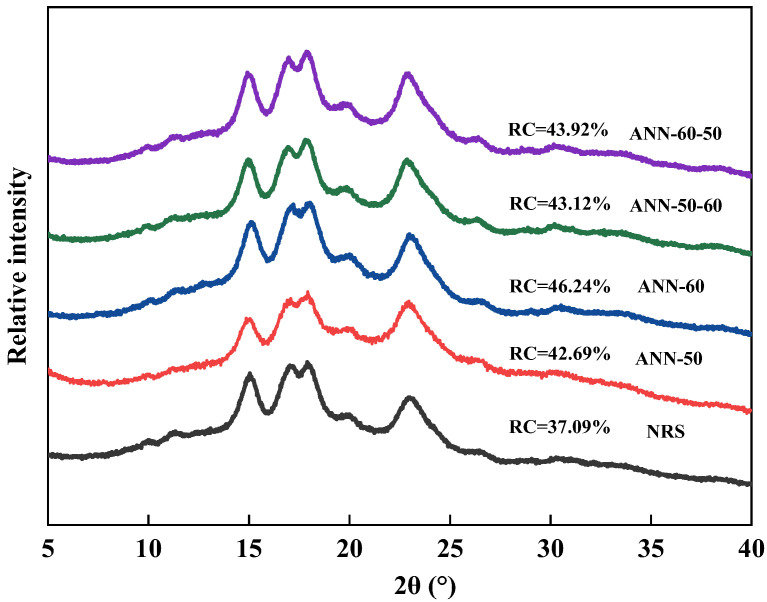
X-ray diffraction patterns of the one and two-step annealed rice starch samples.

The crystallization properties of starch are shown in Figure 2. As shown in the Figure, the natural rice starch had remarkable diffraction peaks at 2θ = 15°, 17°, 18°, and 23° (Figure 2), indicating an A-type crystalline structure [23]. The weak diffraction peak at 2θ = 20° might be associated with starch–lipid complexes in rice starch and correspond to the V-type crystalline structure. The crystalline structure of rice starch did not change before and after annealing, indicating that the double helix structure of the starch did not melt, and the crystalline form was not destroyed during the annealing treatment, which was consistent with the findings of Wang et al. [24].

The relative crystallinity (RC) of natural rice starch was 37.09%, and it significantly increased after the annealing modification, corresponding to the findings of Waduge et al. [25]. The increase in the crystallinity after annealing could be attributed to the interaction of some factors, such as the reduction of defect crystal structure and the formation of new microcrystals [3]. The RCs of ANN−50 and ANN−60 after receiving one-step annealing modification was 42.69% and 46.24%, respectively, increasing by 5.6% and 9.15%, compared with NRS, which may be due to the annealing temperature approaching T_O_ being more conducive to the internal rearrangement of starch molecules and increasing the integrity of starch crystal structure. It was noteworthy that the RCs of the two-step annealing samples were lower than the RC of ANN−60 of the one-step annealing, which may be due to the change in temperature during the two-step annealing not being conducive to the transformation of imperfect crystal structure to perfect crystal structure for starch under hydrothermal action, thereby inhibiting the formation of more new microcrystalline structures.

### 3.3. FT-IR

**Figure 3:**
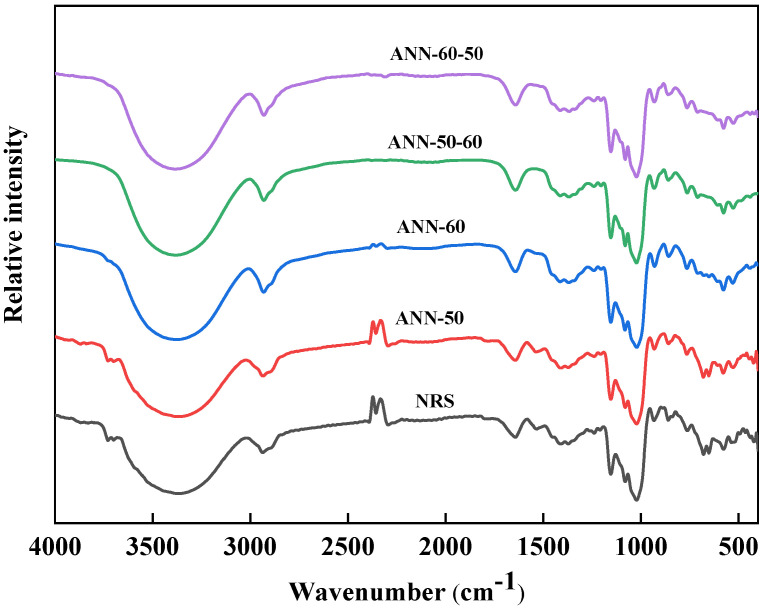
FT-IR spectra of the one and two-step annealed rice starch samples.

The short-range order of rice starch sample molecules was reflected by FT-IR (Figure 3 and Table 1). The bands that appeared in the 1200~800 cm^−1^ region were the embodiments of the stretching and bending vibration information of various chemical bonds, which mainly reflected the stretching vibrations of C−C and C−O and the bending vibrations of C−H−O, and they were sensitive to changes in the short-range ordered structure of starch. As shown in Figure 3 for FT-IR spectroscopy, the annealing treatment had no significant effects on the typical absorption peaks of the natural rice starch, but the strengths of the absorption peaks varied depending on whether the starch was annealing or not, which was consistent with findings of Ji et al. [26], who studied the effects of annealing on three crystal forms of starch.

Absorption strengths at 1047 cm^−1^ and 1022 cm^−1^ were used to characterize the ordered structure of the starch crystalline region and the disordered structure of the amorphous region, respectively, while the absorption strength at 995 cm^−1^ was used to indicate the amount of intramolecular hydrogen bonds of the hydroxyl group [27]. Therefore, R995 cm^−1^/1022 cm^−1^ and R1047 cm^−1^/1022 cm^−1^ are usually used to characterize the double helix structure and order of starch [28]. After the one-step annealing treatment, the ratio of R995 cm^−1^/1022 cm^−1^ increased, which was more significant in ANN−60 than that in ANN−50, indicating that annealing at 60°C was more conducive to the increase in the double helix structure. After the two-step annealing, the ratios of R995 cm^−1^/1022 cm^−1^ and R1047 cm^−1^/1022 cm^−1^ were significantly lower than that of natural starch and one-step annealed starch, indicating a reduced order of starch crystals, which may be due to the fact that the temperature change will disturb the rearrangement of starch molecular chains, thereby reducing the order of starch crystals. In addition, Vela et al. [29] found that the R1047 cm^−1^/1022 cm^−1^ of the rice flour samples remained unchanged at annealing temperatures of 20 °C, 40 °C, and 50 °C, and only the annealing temperature of 60 °C caused a significant difference from the control group, which might be related to partial gelatinization during treatment.

### 3.4. RVA

**Figure 4:**
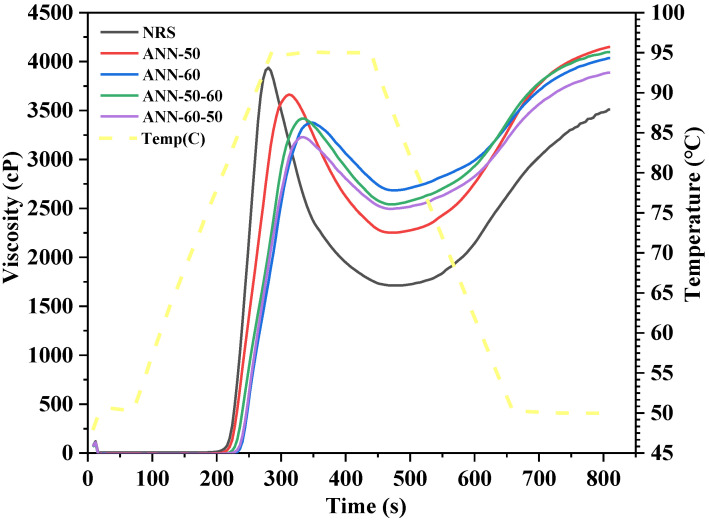
Viscosity profiles of the one and two-step annealed rice starch samples.

The gelatinization characteristic curve and corresponding parameters of rice starch before and after annealing are presented in Figure 4 and Table 2. According to Figure 4, the peak viscosity of starch after annealing decreased by varying degrees, which was consistent with the findings of Shi et al. [2]. The internal crystallization of starch increased, and the interactions between the starch chains were strengthened after annealing, thereby inhibiting the formation of hydrogen bonds between starch and water, reducing the degree of hydration in the amorphous region and limiting the swelling capacity, resulting in a decrease in peak viscosity. ANN−60 that underwent one-step annealing had a more significant reduction of peak viscosity and hydratability of the amorphous region due to the greater hydrothermal intensity applied on starch granules through annealing at temperatures approaching gelatinization. The peak viscosity of ANN−60−50 was significantly lower than that of ANN−50−60 in two-step annealing, which may be due to the fact that the crystal integrity of the starch granules undergoing one-step annealing had changed, and the hydrothermal intensity effect of the subsequent annealing temperature may be smaller.

The breakdown is related to the thermostability and shear stability of the starch granules. Compared with NRS, the breakdown of annealed starch was reduced to varying degrees, which showed that annealing was conducive to increasing the thermostability and shear stability of starch. That is mainly because intramolecular imperfect crystallization in rice starch will form a tighter perfect crystal structure through molecular rearrangement under hydrothermal action, resulting in a more stable structure of the starch [1,8]. The retrogradation value is associated with a short-term retrogradation of starch, with a decrease in the retrogradation value of the starch after annealing, which is usually related to the amylose content and the rearrangement of the amylose leached in gelatinization. The increasing of the final viscosity indicates that the ability of rice starch to form a gel by cooling increases after annealing [30].

### 3.5. DSC

**Figure 5:**
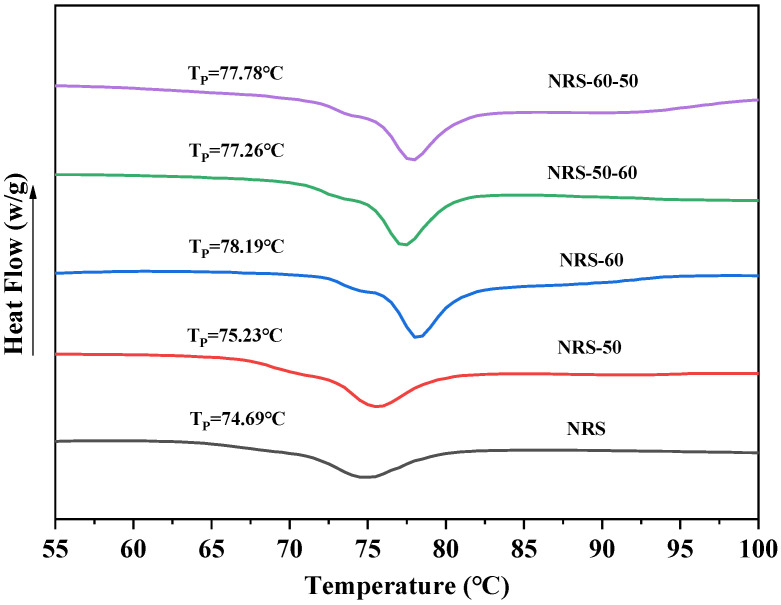
Thermodynamic properties of the one and two-step annealed rice starch samples.

Thermal properties such as T_O_, T_P_, and T_C_ for starch are related to the crystallization degrees and particle sizes of the starch granules. The tighter and more orderly the association between starch molecules is, the more energy is required to destroy its inherent structure in the gelatinization process and the higher the gelatinization temperature is. The effect of annealing treatment on the thermal properties of rice starch is shown in Table 3 and Figure 5. The annealed starch increased in To and T_P_, decreased in T_C_−T_O_, and had no significant change in enthalpy value. It indicates that annealing improves the stability and uniformity of the crystals inside the starch molecules and forms a tighter crystalline structure than the natural starch, resulting in an increase in its gelatinization temperature [13]. This result was consistent with the findings of Waduge et al. [25], who reported that annealing increased the T_O_, T_P_, and Tc of barley starch and decreased the gelatinization temperature range (T_C_−T_O_), attributing this change to the formation of a new double helix structure due to the improvement of the crystal structure and the interaction between amylose–amylose or amylose–amylopectin.

In the two-step annealing, ANN−60−50 had a higher gelatinization temperature than ANN−50−60, which may be because T_O_ of starch was increased by annealing treatment in the first step. Combined with the one-step annealed rice starch in which T_O_, T_P_, and T_C_ of ANN−60 were significantly higher than ANN−50, it was shown that starch was getting higher and higher in intermolecular association and tighter and tighter in crystallization structure when the annealing temperature was getting closer to the gelatinization temperature of the starch. Significantly, the DSC spectrum began to gradually form a multimodal morphology after annealing, which may be due to the fact that part of the new double helix structure formed in the phase change process, since part of the imperfect crystallization transforms into perfect crystallization in starch during annealing. It gradually generated double peaks under heating and melting because of the difference in stability of that part of the structure and the inherent thermostability of starch.

### 3.6. Swelling Power and Solubility

**Figure 6:**
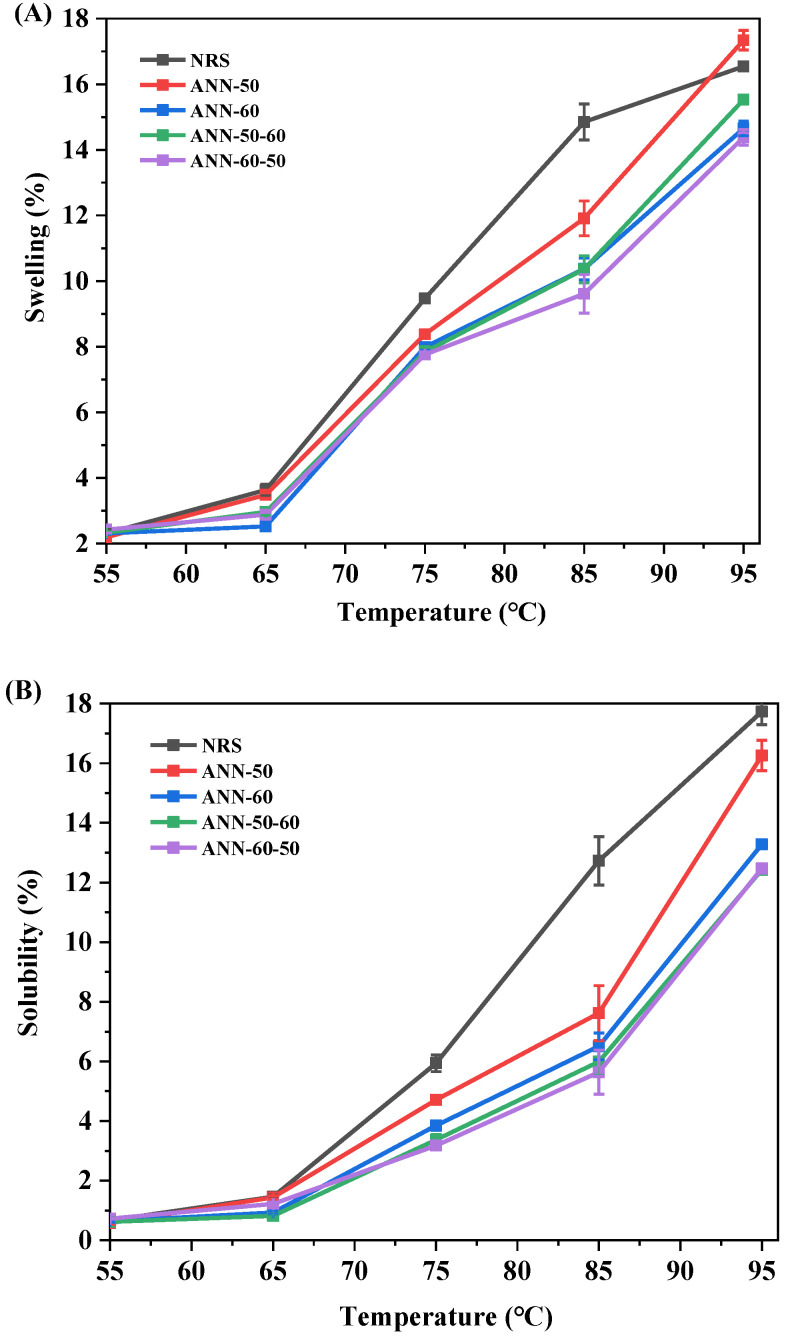
(**A**) Swelling power. (**B**) Solubility index patterns of the one and two-step annealed rice starch samples.

The solubility and swellability of starch samples were measured in 55 °C−95 °C, as shown in Figure 6. The solubility and swellability of starch increased with the rise of temperature before and after annealing. This may be because the gradual absorption and expansion of starch granules with the increase in temperature lead to the accelerated dissolution of amylose, resulting in the increased swellability of starch granules. After annealing, the solubility and swellability of rice starch showed a decreasing trend, which was consistent with findings of Xu et al. [16]. This may be because an increase in crystal integrity and amylose polymerization or interaction reduces the hydration of the amorphous region of starch, thus inhibiting the swelling of starch granules. In addition, a decrease in swellability of the annealed starch showed that the breakage of the amylopectin chain in the annealing process reduced the water-holding capacity of swollen granules [13,25].

During the one-step annealing, the sample ANN−50 had a significantly higher solubility and swellability than ANN−60. When annealing at 60 °C, the imperfect crystallization of starch formed perfect crystallization through unwinding, and the crystallinity increased significantly, compared with that annealed at 50 °C, thereby achieving stronger thermostability, as well as relatively lower solubility and swellability. The two-step annealed rice starch had significantly lower solubility than the one-step annealed rice starch, which may be related to the gradual improvement of starch microcrystals and the enhanced intermolecular interaction of amylose during the annealing process. This change formed a more stable configuration and reduced the leaching of amylose. Meanwhile, the decrease in the swelling power of annealed starch was consistent with the decrease in gelatinization viscosity.

### 3.7. In Vitro Digestibility

**Figure 7:**
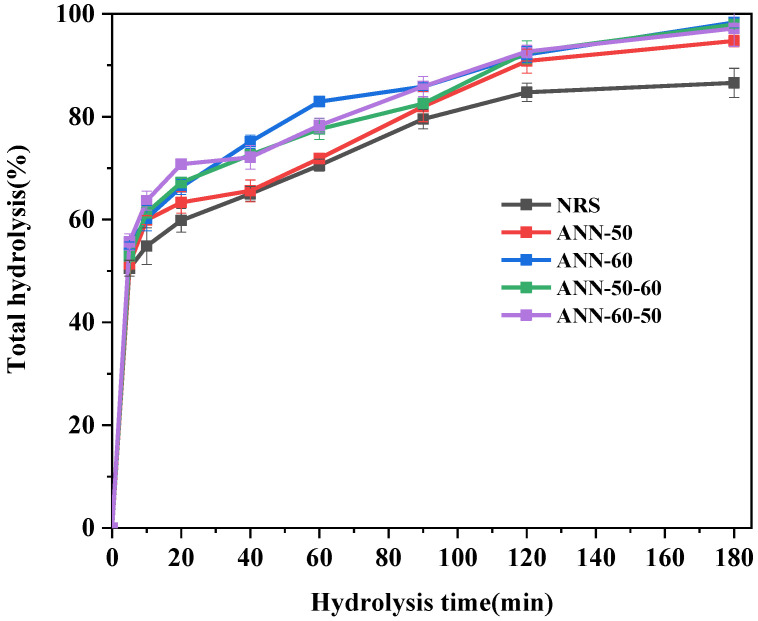
The digestion curve of the one and two-step annealed rice starch samples.

The digestion curves of rice starch before and after annealing are shown in Figure 7. All samples were rapidly digested within 20 min, and starch hydrolysis slowly increased within 20–180 min. Annealing treatment changed the digestibility of starch. Both the one-step annealing and the two-step annealing improved RDS and SDS contents and reduced RS content (Table 4). The one-step-annealed modified ANN-60 was higher than ANN-50. Combined with SEM analysis, the hydrothermal effect was more intense at the annealing temperature of 60 °C, and the surface folds of starch granules increased; thus, hydrolase was more likely to go into starch granules, thereby increasing RDS content and reducing RS.

The increase in ANN-60-50 was most significant in the two-step annealing, with RDS and SDS increasing by 13.2% and 0.12% over NRS and RS content reducing by 12.57%. Combined with SEM analysis, the increase in RDS content and the decrease in RS content may be due to the folds generated on the surface layer of the annealed rice starch, thereby increasing enzyme sensitivity and accelerating the digestion of rapid digestion starch (RDS). The increase in SDS may be due to the annealing process enhancing the strong interaction between the intermolecular chains of starch, which limits the effect of enzymes on the starch molecular chains to some extent [31]. Song et al. [32] annealed starch from potatoes and sweet potatoes and obtained consistent results, with increases in RDS and SDS contents and a decrease in RS content. Since digestive enzymes easily attack amorphous regions and molten crystal defects, the loss of the α-helix structure following partial gelatinization of the annealed starch can explain the increase in SDS and the decrease in RS.

## 4. Conclusions

The results showed that the surface folds of rice starch granules increased, and the Maltese cross was more obvious after annealing. The annealing modification increased the hydration of the amorphous region and crystallization integrity for starch, resulting in decreases in its breakdown, peak viscosity, and retrogradation value and some increase in To and T_P_, which also showed an improvement of the thermostability of rice starch. FTIR showed that no functional groups and new chemical bonds were produced after different annealings of rice starch; the pretreatment prior to two-step annealing increased the T_O_ of starch, and the change of annealing temperature destroyed the natural crystallization integrity, reducing the short-range order of rice starch molecules. Therefore, when starch is modified by annealing, it is important to keep the temperature of the hydrothermal treatment consistent. This is more conducive to the rearrangement of starch molecular chains and the formation of a perfect crystalline structure. The obtained result may be helpful for the development of annealing modified starch with appropriate applications.

## Figures and Tables

**Table 1:** The ratios of 1047 cm^−1^/1022 cm^−1^ and 995 cm^−1^/1022 cm^−1^ of the one and two-step annealed rice starch samples.

Samples	IR (995/1022)/cm^−1^	IR (1047/1022)/cm^−1^
NRS	0.602 ± 0.009 ^c^	0.770 ± 0.007 ^b^
ANN-50	0.627 ± 0.007 ^b^	0.788 ± 0.004 ^a^
ANN-60	0.657 ± 0.004 ^a^	0.777 ± 0.002 ^ab^
ANN-50–60	0.584 ± 0.014 ^d^	0.741 ± 0.003 ^c^
ANN-60–50	0.587 ± 0.023 ^cd^	0.744 ± 0.014 ^c^

Same lowercase letter in the same column indicates non-significant difference, different indicates significant difference (*p <* 0.05).

**Table 2:** Viscosity properties of the one and two-step annealed rice starch samples.

Sample	PV/cP	BD/cP	FV/cP	SB/cP
NRS	3876.67 ± 70.61 ^a^	2182.00 ± 65.94 ^a^	3508.00 ± 14.73 ^d^	1813.00 ± 15.01 ^a^
ANN−50	3723.00 ± 51.12 ^b^	1446.00 ± 29.44 ^b^	4174.00 ± 22.61 ^a^	1897.00 ± 5.00 ^a^
ANN−60	3349.50 ± 38.89 ^c^	716.50 ± 33.23 ^d^	3987.00 ± 70.71 ^b^	1354.00 ± 1.14 ^c^
ANN−50−60	3411.00 ± 9.90 ^c^	853.00 ± 23.52 ^c^	4050.00 ± 63.15 ^b^	1543.00 ± 16.64 ^b^
ANN−60−50	3158.67 ± 63.96 ^d^	810.00 ± 67.98 ^cd^	3863.00 ± 39.89 ^c^	1468.50 ± 106.77 ^b^

Same lowercase letter in the same column indicates non-significant difference, different indicates significant difference (*p <* 0.05).

**Table 3:** Thermodynamic properties of the one and two-step annealed rice starch samples.

Sample	T_O_ (°C)	T_P_ (°C)	T_C_ (°C)	T_C_−T_O_ (°C)	△H (J/g)
NRS	64.42 ± 0.46 ^e^	74.69 ± 0.31 ^e^	81.13 ± 1.10 ^abc^	16.71 ± 1.16 ^a^	9.53 ± 0.12 ^a^
ANN−50	67.16 ± 0.40 ^d^	75.23 ± 0.26 ^d^	80.45 ± 0.45 ^c^	13.29 ± 0.42 ^b^	10.15 ± 0.95 ^a^
ANN−60	72.45 ± 0.23 ^a^	78.19 ± 0.03 ^a^	82.01 ± 0.23 ^a^	9.56 ± 0.39 ^c^	10.27 ± 0.70 ^a^
ANN−50 − 60	70.99 ± 0.10 ^c^	77.26 ± 0.07 ^c^	80.97 ± 0.34 ^bc^	9.98 ± 0.25 ^c^	10.42 ± 0.63 ^a^
ANN−60−50	71.80 ± 0.20 ^b^	77.78 ± 0.07 ^b^	81.82 ± 0.29 ^ab^	10.02 ± 0.13 ^c^	10.31 ± 0.96 ^a^

Same lowercase letter in the same column indicates non-significant difference, different indicates significant difference (*p <* 0.05).

**Table 4:** The amount of RDS, SDS, and RS of the one and two-step annealed rice starch samples.

Samples	RDS	SDS	RS
NRS	51.25 ± 0.59 ^e^	24.16 ± 0.24 ^b^	23.84 ± 0.23 ^a^
ANN−50	53.98 ± 0.12 ^d^	29.32 ± 1.96 ^a^	17.85 ± 0.46 ^b^
ANN−60	56.33 ± 0.12 ^c^	27.13 ± 1.76 ^ab^	16.53 ± 1.87 ^b^
ANN−50−60	59.21 ± 0.12 ^b^	27.99 ± 0.36 ^a^	12.80 ± 0.24 ^c^
ANN−60−50	64.45 ± 0.25 ^a^	24.28 ± 0.37 ^b^	11.27 ± 0.12 ^c^

Same lowercase letter in the same column indicates non-significant difference, different indicates significant difference (*p <* 0.05).

## Data Availability

The data presented in this study are available on request from the corresponding author.
